# Role of insulin resistance in atherosclerosis: underlying mechanisms and emerging stem cell-based therapeutic strategies

**DOI:** 10.3389/fmed.2026.1951400

**Published:** 2026-10-06

**Authors:** Qiuwang Zhang, Yingen Chen, Di Wang, Sandra Pereira, Michael J. B. Kurtyk

**Affiliations:** 1 Division of Cardiology, Keenan Research Center for Biomedical Science, St. Michael's Hospital, Unity Health Toronto, University of Toronto, Toronto, ON, Canada; 2 Department of Cardiology, the First Affiliated Hospital of Henan Medical University, Xinxiang, Henan, China; 3 Department of Endocrinology, the First Affiliated Hospital of Henan Medical University, Xinxiang, Henan, China; 4 Department of Physiology, University of Toronto, Toronto, ON, Canada; 5 Schizophrenia Division, Centre for Addiction and Mental Health, Toronto, ON, Canada

**Keywords:** atherosclerosis, dyslipidemia, endothelial dysfunction, exosome, inflammation, insulin resistance, mesenchymal stem cell, vascular remodeling

## Abstract

Atherosclerosis, a chronic inflammatory disease of the arteries, is the primary cause of ischemic heart disease and stroke, which are the leading drivers of global mortality and major contributors to disability. Important risk factors for atherosclerosis include insulin resistance (IR), hypertension and hyperlipidemia. IR-induced endothelial dysfunction, inflammation, dyslipidemia, vascular remodeling, and prothrombotic effects represent central mechanistic links between IR and atherosclerosis. In this review, these mechanisms are summarized and discussed by incorporating emerging mechanistic insights into IR-induced trained immunity, tissue-selective IR, and mitochondrial dysfunction, with an emphasis on their contributions to atherosclerosis. Furthermore, emerging therapeutic strategies using mesenchymal stem cells (MSCs) and their exosomes to improve IR are discussed, with a focus on their underlying mechanisms, as well as current challenges and outstanding issues in the field. While the current literature shows promising effects of MSCs and their exosomes in improving IR in cellular and animal models, clinical evidence remains limited. To harness the potential therapeutic benefits of MSCs and their exosomes, standardized manufacturing protocols and optimal routes of administration and dosing need to be determined.

## 1 Introduction

Atherosclerosis, a chronic inflammatory disease of the arteries, is the primary cause of ischemic heart disease and stroke, which are the leading drivers of global mortality and major contributors to disability (1, 2). Important risk factors for atherosclerosis include insulin resistance (IR), hypertension and hyperlipidemia (1, 3, 4). Atherosclerosis begins with endothelial injury or dysfunction (5, 6), often triggered by risk factors such as hyperlipidemia, hypertension, and IR (7). Early in the process, reduced nitric oxide (NO) bioavailability and increased oxidative stress increase endothelial permeability, allowing circulating apoB containing lipoproteins, particularly low-density lipoprotein (LDL) to enter the arterial intima (7, 8). Within the arterial wall, LDL undergoes oxidative modification to form oxidized LDL (oxLDL), a highly pro-inflammatory molecule that promotes monocyte recruitment. These monocytes subsequently differentiate into macrophages, internalize oxLDL, and transform into lipid laden foam cells (8, 9). As atherosclerosis progresses, activated macrophages release pro-inflammatory mediators (for example IL-1β, IL-6, TNF-α, and chemokines) that recruit immune cells, intensify local inflammation, and sustain plaque growth and progression (10). In addition, inflammatory mediators within the atheromatous plaque stimulate the migration of vascular smooth muscle cells (VSMCs) from the media into the intima, where these cells proliferate and synthesize extracellular matrix that promotes plaque growth and fibrous cap formation (11).

Insulin, a hormone produced by pancreatic β cells, is well-known to regulate glucose, protein, and lipid metabolism. For example, insulin stimulates glucose uptake in muscle and adipose tissue and suppresses hepatic glucose production to maintain blood glucose homeostasis. Moreover, insulin also modulates cardiovascular function as well as atherogenesis, and these actions can be independent of insulin's metabolic effects (12). Although the effects of insulin on skeletal muscle, adipose tissue, and the liver have been studied extensively, insulin receptors are also expressed in many other tissues, including the endothelium, vascular smooth muscle, and macrophages (13–15). Insulin acts via binding to the insulin receptor, a receptor tyrosine kinase on the plasma membrane of target cells. Upon insulin binding, the receptor is activated, leading to the recruitment of insulin receptor substrates and subsequent activation of downstream signaling cascades, including the phosphoinositide 3 kinase (PI3 K)/Akt pathway and the mitogen activated protein kinase (MAPK) pathway. The PI3 K/Akt pathway primarily mediates metabolic effects, whereas the MAPK pathway mainly regulates mitogenic responses (16).

IR refers to a metabolic condition in which cells fail to respond adequately to insulin, resulting from defects in insulin signaling and other cellular processes that impair insulin action (17, 18). It is a characteristic of type 2 diabetes and often associated with obesity (19, 20). Other mechanisms contributing to IR include endoplasmic reticulum (ER) stress and activation of the unfolded protein response (21), gut microbiota-driven metabolic endotoxemia involving lipopolysaccharide (LPS)/TLR4 signaling (22), and ceramide-based lipotoxicity (23). Cellular senescence has also emerged as an additional contributor to metabolic dysfunction and IR, with evidence linking senescent cells and their senescence-associated secretory phenotype to impaired insulin sensitivity (24). Chronic low-grade inflammation in adipose tissue and liver contributes to IR by increasing pro-inflammatory cytokines such as TNF-α and IL-6 that interfere with insulin signaling (25–28). Lipid accumulation in the liver and skeletal muscle also contributes to IR through the production of metabolites such as diacylglycerol that result in insulin signaling impairments. This ectopic lipid accumulation can result from insulin resistance of lipid metabolism in adipose tissue, namely impaired insulin-induced suppression of lipolysis. Mitochondrial dysfunction and oxidative stress further exacerbate these abnormalities by impairing cellular energy metabolism (26). The mechanisms underlying IR often depend on the tissue type and disease state. There is also the concept of selective IR, where one intracellular pathway emanating from the insulin receptor is dampened, but other intracellular signaling pathways triggered by insulin remain intact. Selective IR also applies to atherogenesis because evidence indicates that impairments in the PI3 K pathway (as opposed to impairments in the MAPK pathway) among local cells are proatherogenic (29).

IR-induced endothelial dysfunction, inflammation, dyslipidemia, vascular remodeling, and prothrombotic effects represent central mechanistic links between IR and atherosclerosis. In this article, these mechanisms are reviewed and summarized with the incorporation of emerging mechanistic insights into IR-induced trained immunity, tissue-selective IR, and mitochondrial dysfunction and their contributions to atherosclerosis. Furthermore, emerging therapeutic strategies using mesenchymal stem cells and their exosomes to improve IR, their underlying mechanisms, and the challenges and outstanding issues in this field are discussed.

## 2 Literature search

Literature for this narrative review was identified through searches of PubMed, Scopus, and Google Scholar using the terms “insulin signaling” or “insulin resistance” in combination with “atherosclerosis”, “endothelial dysfunction”, or “cardiovascular disease”. The primary literature search was restricted to articles published between 2000 and 2026. However, earlier studies identified through manual screening of reference lists were also included when considered foundational or directly relevant to the review objectives. Selection of articles was based on relevance to the review objectives.

## 3 Role of IR in atherosclerosis

IR contributes to the development of atherosclerosis, particularly in individuals with type 2 diabetes and metabolic syndrome. It promotes atherosclerosis through multiple interconnected mechanisms (Figure 1).

**Figure 1:**
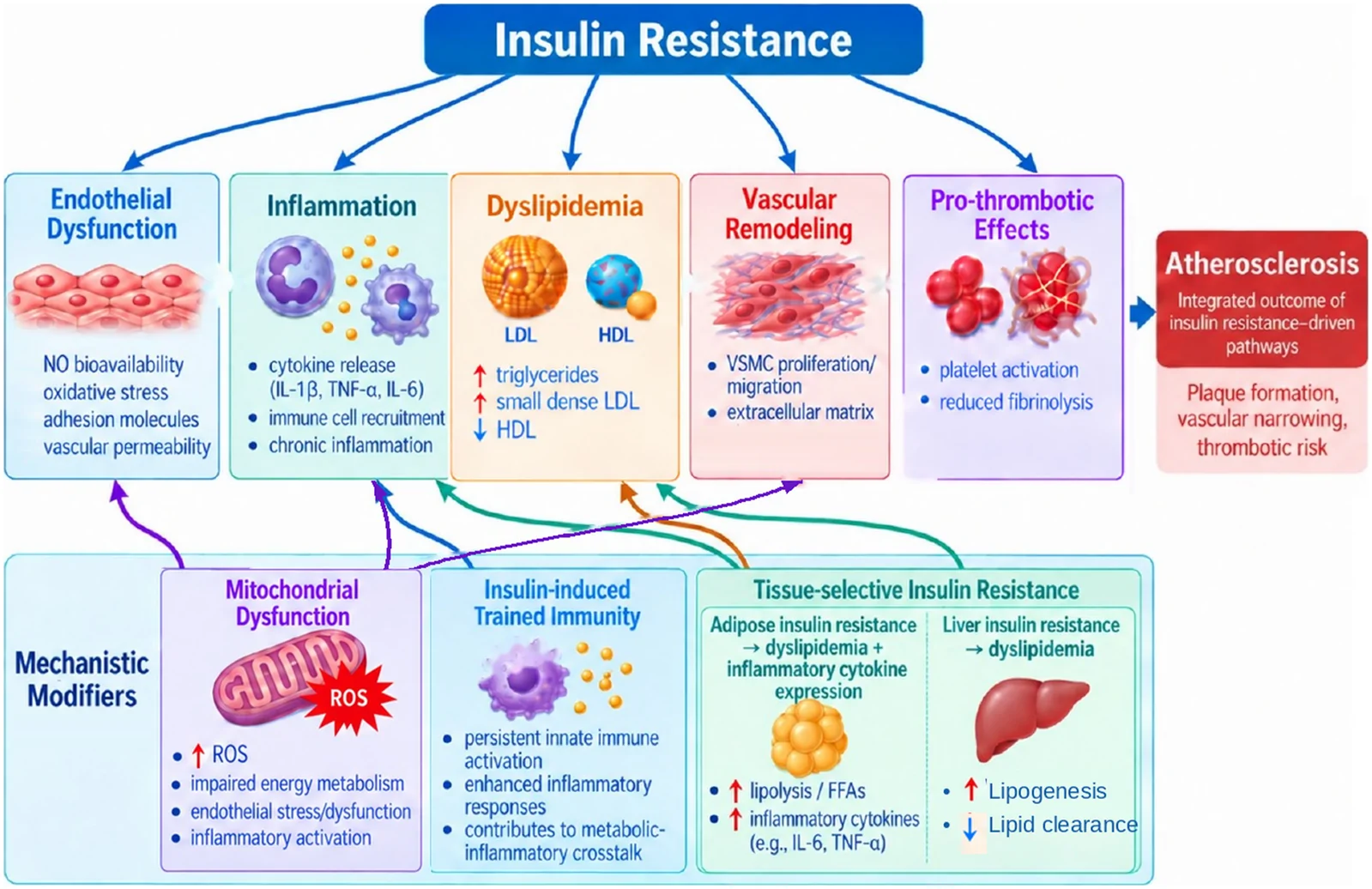
Underlying mechanisms of insulin resistance (IR) contributing to atherosclerosis. IR induces endothelial dysfunction, inflammation, dyslipidemia, vascular remodeling, and a prothrombotic state, which interact to promote vascular inflammation, plaque development, and atherosclerosis. As important mechanistic modifiers, mitochondrial dysfunction is involved in endothelial dysfunction, inflammation, and vascular remodeling, while trained immunity promotes inflammation; hepatic IR contributes to dyslipidemia, and adipose tissue IR contributes to both dyslipidemia and inflammation.

### 3.1 Endothelial dysfunction

Endothelial nitric oxide synthase (eNOS) catalyzes the production of NO, a critical signaling molecule that maintains endothelial homeostasis through its vasodilatory, anti-inflammatory, anti-oxidative, and antithrombotic effects (30, 31). Insulin normally activates eNOS via the PI3 K/Akt pathway to promote NO production in endothelial cells; however, this signaling is impaired under IR conditions (32, 33). Mice fed a high fat diet exhibit IR that is associated with reduced activation of Akt and eNOS, increased lipid deposition, increased expression of the leukocyte adhesion molecule E-selectin, and elevated reactive oxygen species (ROS) production in the aorta (34). E-selectin plays a key role in endothelial inflammation by mediating leukocyte tethering and rolling along the endothelium, while ROS contributes to oxidative stress. Notably, these effects as well as inflammation are diminished by enhanced PI3 K/Akt signaling in murine aorta and human umbilical vein endothelial cells (34).

Duncan et al. developed a mouse model of endothelial-specific IR by the targeted overexpression of a dominant-negative mutant human insulin receptor. They demonstrated that these mice had significant endothelial dysfunction, as shown by blunted aortic vasorelaxation responses to acetylcholine (ACh), an endothelium-dependent, NO-mediated vasodilator and calcium ionophore, compared with wild-type littermates (35). Moreover, increased superoxide generation was detected in coronary microvascular endothelial cells isolated from the mutant mice compared with those from wild-type controls (35). In spontaneously hypertensive rats, IR is associated with reduced NO production in mesenteric artery endothelial cells and attributed to impaired PI3 K/Akt signaling (36). A study in humans assessed IR and brachial artery flow mediated dilation (FMD), a validated measure of endothelial function, and found that subjects with IR exhibited significantly lower FMD than insulin sensitive controls, consistent with impaired nitric oxide mediated vasodilation. This study also demonstrated a significant association between worsening IR and reduced endothelial function across the cohort (37). Chen et al. evaluated endothelial dysfunction and IR in type 2 diabetes and demonstrated significantly reduced forearm blood flow in response to acetylcholine (ACh) in patients with type 2 diabetes and IR relative to control subjects (38). Similar results are reported in numerous clinical studies as summarized by Muniyappa et al. (39).

Mitochondrial dysfunction in IR represents another important mechanistic link between IR and endothelial dysfunction. IR can promote mitochondrial dysfunction by increasing mitochondrial ROS generation. Du et al. demonstrated that IR increased endothelial fatty acid oxidation, leading to excessive mitochondrial electron transport chain-derived superoxide production (40). In cultured aortic endothelial cells, increased fatty acid oxidation reduced the activities of eNOS and prostacyclin synthase. Importantly, these abnormalities were also observed in IR Zucker rats and high-fat diet-induced IR mice and were prevented by inhibiting adipose fatty acid release or mitochondrial fatty acid oxidation through carnitine palmitoyltransferase 1, a key regulator of mitochondrial fatty acid oxidation (40). These findings provide direct evidence that altered mitochondrial metabolism contributes to endothelial dysfunction in IR. Dynamin-related protein 1 (Drp1) is a key regulator of mitochondrial fission. *In vitro* inhibition of Drp1 reduced mitochondrial fission, mitochondrial superoxide production, and endothelial dysfunction under high-glucose conditions (41). In streptozotocin (STZ)-induced diabetic *ApoE*^−^/^−^ mice, mitochondrial division inhibitor 1, a potent and selective Drp1 inhibitor, reduced mitochondrial fragmentation, attenuated oxidative stress, and ameliorated endothelial dysfunction and atherosclerotic lesion formation (41). Other *in vitro* and *in vivo* studies have also shown that hyperglycemia, a condition closely associated with IR, activates Drp1 in endothelial cells, thereby promoting mitochondrial fragmentation, increasing mitochondrial ROS production, and contributing to endothelial dysfunction (42–44).

### 3.2 Inflammation

Animal studies provide strong mechanistic evidence that IR promotes chronic vascular and systemic inflammation that accelerates atherosclerosis. In *ApoE*^−^/^−^ mice with endothelial cell specific insulin receptor knockout leading to IR, endothelium-dependent vasodilation was impaired and endothelial cell expression of endothelium vascular adhesion molecule-1 (VCAM-1) was increased (45). VCAM-1 is an endothelial adhesion molecule that facilitates leukocyte adhesion and transmigration into the arterial intima, promoting early vascular inflammation and atherogenesis. Indeed, compared with controls, insulin receptor knockout mice exhibited significantly greater adhesion of mononuclear cells to the endothelium, consistent with enhanced vascular inflammation and accelerated progression of atherosclerosis (45). The PI3 K/Akt pathway is essential for maintaining insulin sensitivity and restraining atherogenesis. *Akt1^−^/^−^ApoE^−^/^−^* mice developed both IR and atherosclerosis and demonstrated significantly increased lesional inflammatory cytokines and decreased eNOS activity compared with *ApoE^−^/^−^* mice, leading to more severe coronary arterial lesions (46). Laskou et al. developed a mouse model of IR and atherosclerosis by feeding *LDLR*⁻/⁻ mice a high-fat, high-cholesterol diet. They reported that platelet depletion reduced plaque macrophage content and attenuated inflammatory gene expression in macrophages in these mice, thereby facilitating resolution of inflammation (47). Consistent with these results, clinical data showed that patients with IR and lower platelet counts had decreased pro-inflammatory signaling pathways in circulating monocytes (47).

IR promotes a pro-inflammatory milieu, contributing to the development and progression of atherosclerosis in humans. Insulin-resistant individuals exhibit elevated circulating inflammatory markers, including C-reactive protein (CRP), interleukin-6 (IL-6), and TNF-α, which are well-established predictors of cardiovascular risk and are also mechanistically involved in atherogenesis (48–50). Adiponectin, produced primarily by adipocytes, is widely regarded as an anti-inflammatory adipokine; it inhibits NF-*κ*B signaling, a central inflammatory pathway involved in atherosclerosis, suppressing TNF-α and IL-6 expression in macrophages and other immune cells (51, 52). Additionally, adiponectin reduces IR (53). Komura et al. showed that VCAM-1 and intercellular adhesion molecule-1 (ICAM-1) were significantly increased in aorta in adiponectin knockout mice compared to wild-type mice after lipopolysaccharide (LPS) treatment (54). Therefore, adiponectin represses VCAM-1 and ICAM-1 expression, limiting leukocyte recruitment to vessel walls and mitigating inflammatory response (54). A systematic review and meta-analysis revealed an inverse association between circulating adiponectin levels and carotid intima media thickness in patients with and without atherosclerosis (55). These findings suggest that adiponectin exerts protective effects against atherosclerosis by mitigating insulin resistance and inflammation.

Dysregulated insulin signaling can promote inflammatory responses in macrophages. Experimental studies have demonstrated impaired insulin/PI3 K signaling in macrophages under diabetic or high-glucose conditions, indicating that macrophages can develop cell-intrinsic IR, thereby promoting inflammatory macrophage activation, including increased expression of proinflammatory cytokines and a shift toward a pro-inflammatory M1-like phenotype (56, 57). Notably, mitochondrial dysfunction may link IR and atherosclerosis through increased mitochondrial ROS production and macrophage inflammation. Ming et al. demonstrated that arginase II promotes mitochondrial superoxide and hydrogen peroxide production in macrophages, thereby enhancing the expression of proinflammatory mediators (IL-6, TNF-α, and MCP-1); inhibition of mitochondrial ROS attenuated these inflammatory responses. In mouse models, arginase II deficiency improved insulin sensitivity and reduced atherosclerotic lesion formation, macrophage inflammation, and necrotic-core size (58). Miao et al. further showed that macrophage-specific deficiency of CGI-58 impaired PPAR*γ*-dependent mitochondrial function, increased ROS production, and activated the NLRP3 inflammasome, thereby enhancing the expression of proinflammatory cytokines including IL-1β. TNF-α, and IL-6, and exacerbating high-fat-diet-induced IR; pharmacological ROS inhibition or NLRP3 silencing attenuated these effects (59). Together, these findings support a role for mitochondrial dysfunction in the development of IR and, in turn, atherosclerosis at least partly by promoting inflammation.

Adipose IR is associated with increased production of pro-inflammatory mediators, which may contribute to systemic inflammation and thereby promote atherosclerosis. Odeniyi et al. reported that treatment of 3T3-L1 adipocytes with TNF-α under hypoxic conditions substantially reduced insulin-stimulated glucose uptake compared with control cells, indicating the development of cellular IR; concomitantly, the expression of the pro-inflammatory mediators MCP-1 and IL-6 was significantly increased in insulin-resistant adipocytes (60). Mammalian target of rapamycin complex 2 (mTORC2), a major component of the insulin signaling pathway, promotes Akt activation, thereby supporting insulin-mediated glucose uptake and metabolic homeostasis (61). Shimobayashi et al. showed that impaired insulin-mTORC2 signaling contributed to IR in 3T3-L1 adipocytes, resulting in increased MCP-1 expression (62). *In vivo* studies in mice with adipose-specific mTORC2 knockout and diet-induced obesity further revealed that adipose IR caused local accumulation of pro-inflammatory macrophages, likely due to increased MCP-1 production in adipose tissue (62). Consistent with these findings, an independent study showed that enhancing insulin-mTORC2 signaling with FGF1 ameliorated adipose IR and reduced its associated inflammatory responses (63).

Emerging evidence suggests that hyperglycemia, a common feature of IR, may contribute to atherosclerosis through persistent reprogramming of innate immune cells (64, 65). In particular, hyperglycemia has been shown to induce trained immunity in monocytes and macrophages that promotes inflammatory responses. Thiem et al. demonstrated that exposure to hyperglycemic conditions induced a persistent pro-inflammatory phenotype in human monocytes and murine macrophages that was retained after normalization of glucose levels (66). Edgar et al. further demonstrated in an *in vitro* cell culture model that high glucose altered the metabolic profile of both primary human monocytes and mouse bone marrow-derived monocytes (BMDMs), characterized by enhanced glycolytic metabolism (67). In parallel, high glucose increased the expression of IL-6, a pro-inflammatory cytokine, and decreased the expression of Ym1 and Fizz1, markers associated with type 2/anti-inflammatory macrophage activation, in BMDMs. These effects were reversed by dichloroacetate, an inhibitor of glycolysis. Dichloroacetate also reduced BMDM adhesion to endothelial cells and ox-LDL uptake under high-glucose conditions. Furthermore, BMDMs isolated from diabetic mice retained a hyperglycemic memory in their gene expression profile even when cultured under normoglycemic conditions, characterized by higher IL-6 expression and lower Ym1 and Fizz1 expression compared with BMDMs from non-diabetic mice (67). This persistent phenotype under physiological glucose conditions is consistent with hyperglycemia-induced trained immunity. *In vivo*, atherosclerosis-prone *LDLR*⁻/⁻ mice transplanted with bone marrow from diabetic mice developed significantly greater atherosclerosis than those receiving bone marrow from normoglycemic mice. Similar transcriptional signatures were identified in circulating leukocytes and atherosclerotic plaque macrophages from patients with diabetes, suggesting a potential role for hyperglycemia-induced trained immunity in human disease (67).

More recently, Vinci et al. extended these findings to hematopoietic stem/progenitor cells (HSPCs), demonstrating that high glucose induced persistent chromatin modifications, including increased H3K4me1 and SETD7 recruitment at the NF-*κ*B p65 promoter, together with increased production of inflammatory cytokines such as IL-6 and TNF-α (68). These epigenetic changes were retained following differentiation of HSPCs into pro-inflammatory monocytes and were also observed in HSPCs from patients with type 2 diabetes (68). Collectively, these findings suggest that hyperglycemia arising in the setting of IR may establish a trained innate immune phenotype through persistent metabolic and epigenetic reprogramming of myeloid cells and their progenitors. This sustained inflammatory phenotype may contribute to vascular inflammation and provide a potential mechanistic link between IR, chronic inflammation, and atherosclerosis.

### 3.3 Dyslipidemia

Animal studies support a causal role for IR in the development of dyslipidemia through hepatic, brain-mediated, and systemic mechanisms (69, 70). Diet induced IR by high fructose and high fat feeding in hamsters is associated with hypertriglyceridemia, hypercholesterolemia, and hepatic lipid accumulation, findings that support a mechanistic link between impaired insulin signaling and lipid dysregulation (70). Brain insulin signaling, particularly within the hypothalamus, has been shown to regulate lipid metabolism by suppressing adipose lipolysis and modulating hepatic triglyceride secretion (71, 72). Therefore, brain IR may contribute to dyslipidemia. Broader reviews of animal models reinforce the evidence that IR drives dyslipidemia via increased hepatic lipogenesis, altered lipoprotein metabolism, and impaired lipid clearance (73).

Many clinical studies have also shown that IR disrupts normal lipid metabolism, contributing to dyslipidemia and the formation of an atherogenic lipid profile. Kelley et al. demonstrated that obese individuals exhibited greater triglyceride accumulation within skeletal muscle than lean controls as a result of reduced fatty acid oxidation. This accumulation contributed to IR and subsequently promoted dyslipidemia (74). Pihlajamäki et al. measured serum cholesterol precursors as markers of cholesterol synthesis and serum cholesterol as a marker of cholesterol absorption in normoglycemic men. They found that those with IR had increased cholesterol synthesis and decreased cholesterol absorption, accompanied by substantially elevated triglycerides but similar levels of LDL-C and HDL-C, compared with insulin-sensitive controls (75). A study of a large cohort of adults with type 2 diabetes has found that patients with IR have significantly higher levels of triglyceride (TG) and LDL-C but lower HDL-C (76). In insulin resistant states, increased adipose tissue lipolysis raises circulating free fatty acid concentrations, promotes hepatic triglyceride synthesis, and contributes to dyslipidemia (73). In a prospective study that included participants with and without cardiovascular disease, lower estimated glucose disposal rates, indicative of IR, were associated with an increased risk of cardiovascular disease, including atherosclerosis. Accordingly, a higher triglyceride glucose index, a surrogate marker of IR, was associated with an increased risk of cardiovascular disease (77). Of interest, a higher metabolic score for insulin resistance (METS-IR) predicted greater severity of coronary artery disease. Similarly, the triglyceride glucose index, triglyceride glucose body mass index (TyG-BMI), and triglyceride to high density lipoprotein cholesterol (TG/HDL-C) ratio, all surrogate markers of IR that reflect triglyceride rich metabolic states, were positively correlated with coronary artery disease severity (78).

In the context of IR-induced dyslipidemia, hepatic and adipose selective IR represent two major tissue-specific contributors. Biddinger et al. reported that liver-specific insulin receptor knockout (LIRKO) mice fed a normal chow diet developed a proatherogenic lipoprotein profile characterized by decreased HDL cholesterol and increased VLDL cholesterol and other apoB-containing lipoproteins (69). Mechanistically, hepatic IR increased apoB-containing lipoprotein secretion while impairing their clearance, in part through reduced LDL receptor expression. When challenged with an atherogenic diet, LIRKO mice developed severe hypercholesterolemia and extensive atherosclerosis, whereas control mice did not (69). These findings demonstrate that hepatic IR alone is sufficient to promote dyslipidemia and significantly increase susceptibility to atherosclerosis. Semple et al. found that patients with a selective postreceptor defect in AKT2 exhibited increased hepatic lipogenesis, hepatic steatosis, triglyceride-enriched VLDL, hypertriglyceridemia, and reduced HDL cholesterol, whereas individuals with generalized IR due to defects in the insulin receptor did not exhibit this dyslipidemic phenotype (79). Smith et al. further demonstrated in humans with obesity-associated nonalcoholic fatty liver disease that hepatic *de novo* lipogenesis was inversely associated with hepatic insulin sensitivity (80). These studies provide evidence supporting a role for increased hepatic lipogenesis in the dyslipidemia associated with hepatic IR. In contrast, Goedeke et al. observed that high-fat-diet-induced hepatic IR *per se* attenuates murine *de novo* lipogenesis (81). Taken together, the current literature indicates that the relationship between hepatic IR and *de novo* lipogenesis is complex and context-dependent.

In contrast, adipose tissue IR contributes to dyslipidemia primarily through impaired insulin-mediated suppression of lipolysis, thereby increasing adipose tissue lipolysis and the release of free fatty acids into the circulation (82–84). Increased free fatty acid flux to the liver provides substrate for triglyceride synthesis and promotes hepatic VLDL production. In addition, adipose tissue IR impairs lipid storage, which may reduce the capacity of adipose tissue to buffer circulating fatty acids, thereby promoting fatty-acid spillover and ectopic lipid delivery to the liver (85). Consistent with these mechanisms, human studies have shown that impaired insulin-mediated suppression of adipose tissue lipolysis is associated with higher circulating triglycerides and other features of atherogenic dyslipidemia (86–88). More recent evidence indicates that adipose IR is independently associated with higher triglycerides and lower HDL cholesterol even after accounting for hepatic IR (86), suggesting that adipose IR contributes to dyslipidemia beyond hepatic IR alone.

### 3.4 Vascular remodeling

During atherogenesis, vascular smooth muscle cells undergo phenotypic activation, proliferate, migrate from the media into the intima, and synthesize extracellular matrix. This results in neointimal thickening and contributes to fibrous plaque formation. NO has been shown to exert a key anti-atherogenic effect by suppressing VSMC proliferation and neointimal formation (89–92). Rudic et al. showed that targeted disruption of endothelial eNOS in mice led to a decrease in NO and an increase in wall thickness accompanied by a hyperplastic response of the arterial wall in the common carotid artery (89). IR in endothelial cells decreases NO bioavailability (13), which removes inhibition on VSMC proliferation and migration, thereby promoting plaque development and progression (89, 90). Mechanistically, NO can react with superoxide to form peroxynitrite, a highly reactive free radical, and both NO and peroxynitrite decrease cell growth and DNA synthesis in cultured VSMCs (91). While NO induces cytostasis in VSMCs by a redox-sensitive mechanism independent of peroxynitrite formation; peroxynitrite induces redox-independent VSMC cytotoxicity and apoptosis (91). Li et al. demonstrated that VSMC-specific insulin receptor knockout in *ApoE*⁻/⁻ mice resulted in larger atherosclerotic plaques characterized by reduced collagen content and expanded necrotic cores, features of unstable plaque, compared with *ApoE*⁻/⁻ controls (14). These findings suggest that insulin receptor deficiency disrupts insulin actions on VSMCs to alter plaque composition, thereby contributing to the formation of unstable atherosclerotic plaques. In addition, higher levels of ICAM-1 and VCAM-1 in VSMCs of arterial walls were observed in insulin receptor and *ApoE* double knockout mice compared with *ApoE*⁻/⁻ mice, indicating that loss of insulin function shifted VSMCs into a more inflammatory profile. Thrombospondin-1(Thbs1) and matrix metallopeptidase 2 (MMP2) were also increased in VSMCs of arterial walls in double knockout mice. Thbs1 was found to upregulate pro-inflammatory cytokines in VSMCs, including ICAM-1, monocyte chemoattractant protein 1, and IL-6 (14). Of note, in cultured VSMCs isolated from insulin receptor and *ApoE* double knockout mice, insulin activated Akt (phospho-AKT) and its downstream transcription factor forkhead box O1 (FoxO1) levels were significantly decreased compared with those from *Apo*E^−^/^−^ mice. Decreased AKT activity increased MMP-2 expression, whereas impaired FoxO1 function upregulated Thbs1 and induced pro-inflammatory cytokine expression. Both alterations contributed to reduced plaque stability (14).

Under normal conditions, insulin signaling through the PI3 K/Akt pathway maintains VSMC quiescence, but in insulin-resistant states this pathway is selectively impaired, while the MAPK pathway remains relatively intact or overactive, promoting VSMC migration and proliferation (92). Wang et al. observed that inhibition of MAPK activation using PD98059, a MAPK selective inhibitor, resulted in decreased VSMC migration (93). In contrast, treatment of VSMCs with wortmannin, a PI3 K/Akt pathway inhibitor, downregulated *α*-smooth muscle actin (*α*-SMA) expression, indicating a switch from the contractile, differentiated phenotype toward a more dedifferentiated, synthetic state, as *α*-SMA is highly expressed in quiescent contractile VSMCs but reduced in activated VSMCs (93). IR is also associated with increased oxidative stress and inflammation, as summarized above. These processes further stimulate VSMC proliferation and extracellular matrix production and promote fibrous plaque formation (93).

Mitochondrial dysfunction may contribute to vascular remodeling in IR-associated atherosclerosis by promoting VSMC migration, proliferation, and phenotypic transformation. In VSMCs exposed to hyperinsulinemia, mitochondrial dysfunction characterized by membrane depolarization, reduced mitochondrial mass, and increased mitochondrial ROS was associated with enhanced VSMC migration and proliferation, suggesting a link between abnormal insulin signaling, mitochondrial dysfunction, and vascular remodeling (94). Ahn et al. reported that mitochondrial dysfunction enhanced the migration of VSMCs via suppression of Akt phosphorylation (95). More recent evidence from a type 2 diabetes mouse model showed that increased mitochondrial ROS and blunted mitochondrial respiration promoted VSMC proliferation and neointima formation, while inhibition of mitochondrial calcium uniporter signaling reduced mitochondrial ROS, preserved mitochondrial respiration, and attenuated both responses (96). In established atherosclerosis, reduced mitochondrial respiration and mitochondrial DNA integrity have also been observed in human plaques and plaque-derived VSMCs; enhancing mitochondrial respiration in *ApoE*^−^/^−^ mice reduced necrotic-core formation and increased fibrous-cap area, indicating that mitochondrial dysfunction can influence plaque remodeling and stability (97). Together, these findings suggest that mitochondrial dysfunction may affect vascular remodeling in IR-associated atherosclerosis, particularly through effects on VSMC behavior and plaque composition.

### 3.5 Prothrombotic effects

Several clinical studies have demonstrated that IR is associated with a prothrombotic state that could contribute to atherosclerosis through sustained platelet activation. IR can develop in patients with type 1 diabetes mellitus, a disease that is caused by insulin deficiency resulting from immune-mediated destruction of pancreatic β-cells. Sagar et al. recently reported that platelets from patients with type 1 diabetes exhibited increased basal activity and hyperactivation following stimulation. Furthermore, patients with IR demonstrated significantly greater basal platelet activation and enhanced responses to stimulation than those without IR (98). Basili et al. reported that individuals with obesity with a lower insulin sensitivity index had a significantly higher level of urinary 11-dehydro-TXB2, a biomarker of platelet activation and thromboxane production. Multiple regression analysis revealed that IR is a major determinant of platelet activation in obesity (99). In another study, De Pergola et al. investigated the association of soluble P-selectin (sP-sel), another indicator of platelet activation, with IR in obese subjects. They found that sP-sel levels were significantly higher in overweight and obese insulin resistant subjects than normal-weight controls (100). IR is also associated with impaired fibrinolysis, characterized primarily by increased plasminogen activator inhibitor 1, thereby potentially promoting a prothrombotic state (101, 102). Therefore, the prothrombotic state associated with IR may contribute to atherosclerosis through platelet activation, acceleration of thrombus formation, and increased risk of plaque rupture, ultimately leading to acute cardiovascular events.

## 4 Heterogeneity of experimental models and clinical measures of IR

Although many of the studies reviewed above suggest that PI3 K/Akt signaling is impaired while MAPK signaling remains relatively preserved or enhanced in IR, experimental and clinical evidence should not be considered a unified body of evidence supporting this signaling paradigm (29, 103). For example, diet-induced animal models of IR reproduce a broader metabolic milieu characterized by hyperglycemia, dyslipidemia, inflammation, and oxidative stress, all of which can influence PI3 K/Akt and MAPK signaling as well as multiple other pathways simultaneously (104, 105). Furthermore, clinical studies typically use HOMA-IR, the triglyceride-glucose (TyG) index, and the triglyceride-to-HDL cholesterol (TG/HDL-C) ratio as surrogate measures of systemic IR rather than directly assessing insulin receptor signaling or PI3 K/Akt and MAPK activity (106, 107). Therefore, associations between these clinical indices and atherosclerotic outcomes should be interpreted as evidence linking systemic IR or its associated metabolic phenotype to cardiovascular risk, rather than as direct confirmation of the PI3 K/Akt-MAPK signaling paradigm. Another issue not addressed in this review but worth noting is that compensatory hyperinsulinemia may occur in IR and may excessively activate the relatively preserved MAPK pathway, while the PI3 K/Akt pathway remains relatively blunted. Therefore, some proatherogenic effects attributed to IR may reflect excessive insulin stimulation of relatively preserved mitogenic signaling pathways. Collectively, these models provide complementary but distinct types of evidence, and the molecular mechanisms linking IR to atherosclerosis should be regarded as multifactorial and context dependent rather than being attributed to any single experimental model, signaling pathway, or surrogate measure of IR.

## 5 An emerging strategy for IR management: mesenchymal stem cell-based approaches

Current approaches to improve insulin IR include lifestyle modification, pharmacological therapy, and, in selected cases, surgical intervention. Lifestyle modifications, such as weight loss, increased physical activity and dietary changes, have been shown to significantly enhance insulin sensitivity and glucose uptake (108). Pharmacological interventions include agents such as metformin, which reduces hepatic glucose production and improves peripheral insulin sensitivity (109); thiazolidinediones are potent insulin sensitizers that improve insulin resistance through activation of PPAR*γ*, but their clinical use has declined because of concerns regarding adverse effects (110). Glucagon like peptide 1 (GLP-1) receptor agonists have demonstrated benefits in reducing insulin resistance in addition to promoting weight loss (111, 112). Sodium glucose cotransporter 2 (SGLT2) inhibitors also improve insulin sensitivity through reduction of glucotoxicity, enhancement of metabolic efficiency, and favorable effects on body weight and adiposity (113). In individuals with severe obesity, bariatric surgery is an effective treatment option that produces rapid and sustained improvements in insulin sensitivity and promotes weight loss (114).

Mesenchymal stem cells (MSCs) and MSC-derived exosomes have been explored as emerging therapeutic strategies for lowering blood glucose and improving IR; exosomes are small extracellular vesicles, typically ∼30–150 nm in diameter, that originate from the endosomal pathway. They carry bioactive molecules, including proteins, lipids, mRNAs, and microRNAs, which can be transferred to recipient cells and modulate cellular signaling and function (115). Although many studies have focused on the effects of MSCs and their exosomes on glycemic control and β-cell function, relatively fewer have specifically investigated their effects on IR (116). Here, we summarize the key evidence regarding the effects of MSCs and their exosomes on IR.

MSCs and their exosomes derived from various sources, including umbilical cord, bone marrow, and adipose tissue, have been investigated in both cellular and animal models (117–126). The results suggest that MSCs and exosomes exert effects through pleiotropic mechanisms, including restoration of insulin sensitivity by activating the PI3 K/AKT pathway, suppression of pro-inflammatory cytokine production, immunomodulation, and gene regulation (Figure 2). Kim et al. reported that conditioned medium from human umbilical cord-derived MSCs improved IR in palmitate-induced insulin-resistant C2C12 skeletal muscle cells. Mechanistically, the conditioned medium enhanced insulin signaling, as evidenced by increased AKT phosphorylation and translocation of glucose transporter 4 to the cell membrane, thereby improving glucose uptake (122). Human umbilical cord-derived MSCs also ameliorate IR by suppressing NLRP3 inflammasome-mediated inflammation and reducing the expression of IL-1β and TNF-α in type 2 diabetes rats (123). Xie et al. demonstrated that human umbilical cord-derived MSCs promoted macrophage polarization toward an anti-inflammatory phenotype, thereby improving IR in diabetic rats (124). Bone marrow derived SMCs decreases blood glucose levels and improves IR in type 2 diabetic rats, which is associated with upregulation of glucose transporter 4 and increased phosphorylation of insulin receptor substrate 1 and AKT in the liver, skeletal muscle, and adipose tissue (125). Cao et al. reported that adipose-derived MSCs reduced blood glucose levels and ameliorate IR in high-fat diet-induced obese mice, which were accompanied by upregulation of peroxisome proliferator-activated receptor *γ* (PPAR*γ*) and the insulin receptor and downregulation of IL-6 in the liver (126).

**Figure 2:**
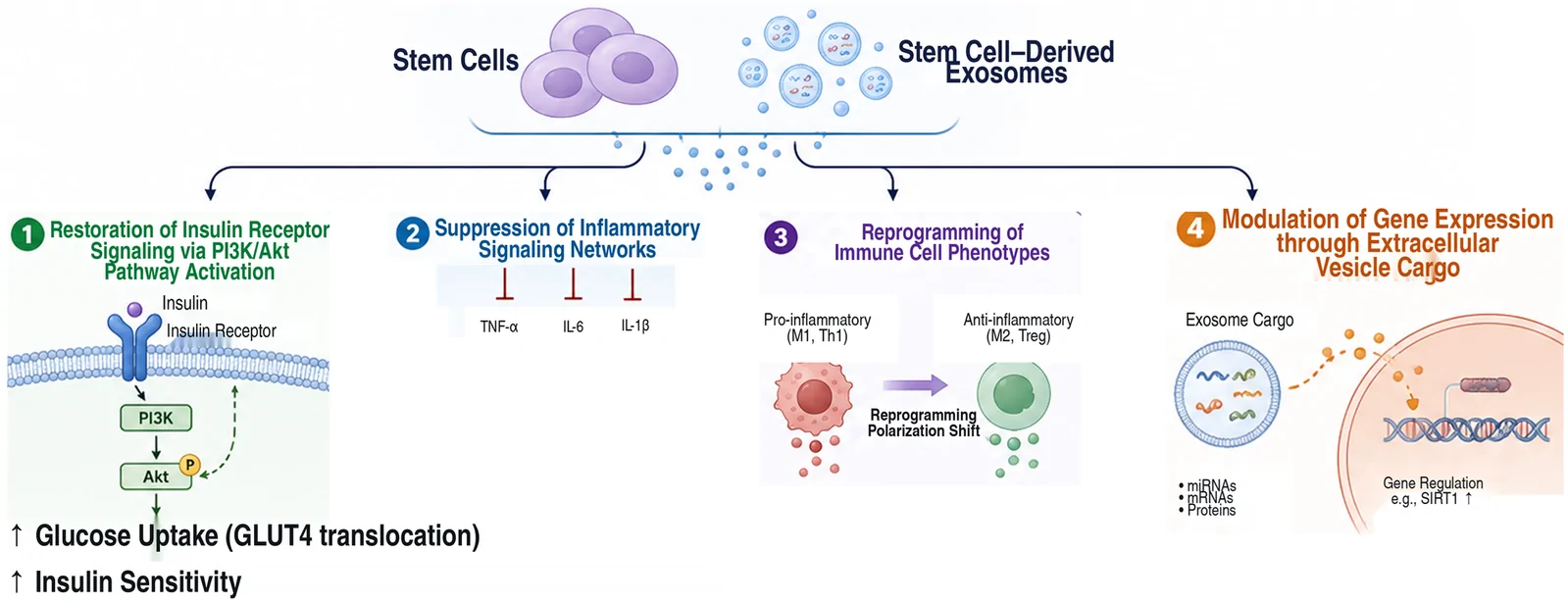
Stem cells and their exosomes for improving insulin resistance. Stem cells and exosomes improve insulin resistance through pleiotropic mechanisms, including the activation of the PI3 K/AKT pathway to restore insulin signaling, suppression of inflammatory cytokine production, immunomodulation, and extracellular vesicle mediated regulation of gene expression to reprogram metabolic pathways to increase insulin sensitivity.

MSC-derived exosomes have been investigated for their ability to ameliorate IR and offer several advantages as a cell-free therapeutic approach, including lower risks associated with viable cell administration, reduced immunogenicity, and greater potential for standardized storage and dosing. Chen et al. showed that exosomes isolated from human umbilical SMCs upregulated the expression of sirtuin-1 and insulin receptor substrate-1 in insulin resistant human adipocytes, thereby enhancing cellular glucose uptake and improving insulin sensitivity (117). SIRT1, an NAD⁺-dependent deacetylase, promotes insulin sensitivity by positively regulating insulin signaling, suppressing NF-*κ*B-mediated inflammation, and maintaining mitochondrial metabolic function (127, 128). Reduced SIRT1 activity has been implicated in the development of IR and associated metabolic dysfunction (129). Of interest, Su et al. observed increased expression of miR-29b-3p in exosomes released from bone marrow-derived MSCs isolated from aged IR mice (121). miR-29b-3p targets and inhibits SIRT1; introduction of a miR-29b-3p inhibitor into BM-MSCs upregulated SIRT1 expression, and infusion of these cells significantly ameliorated IR in aged mice. In contrast, infusion of BM-MSCs overexpressing miR-29b-3p induced IR in young mice (121). Exosomes from mouse bone marrow MSCs have been shown to improve metabolic homeostasis in high-fat diet obese mice, including reduction of obesity, inhibition of M1-type proinflammatory factor expression, and improvement of insulin sensitivity. *In vitro* experiments using insulin resistant 3T3-L1 adipocytes demonstrated that exosomes from bone marrow MSCs enhanced PI3 K/AKT signaling and upregulated glucose transporter protein 4 (118).

Despite promising effects of MSCs and their exosomes in improving IR in cellular and animal models, clinical evidence regarding their effects on IR remains limited. Hu et al. evaluated human Wharton's jelly-derived MSCs for the treatment of type 2 diabetes and observed a significant reduction in blood glucose levels, but no significant improvement in HOMA-IR following cell transplantation (130). A similar lack of improvement in HOMA-IR was observed in patients with type 2 diabetes after transplantation of human umbilical cord-derived MSCs (131). Other studies reported insulin independence in patients with diabetes following MSC treatment; however, these studies did not report direct evidence of improved IR assessed by HOMA-IR or other measures of IR (132, 133). Of note, all these studies were open-label and included relatively small sample sizes, ranging from 6 to 31 participants. Although larger, randomized, controlled clinical trials are warranted to determine whether MSC therapy can improve IR and potentially reduce the associated risk of atherosclerosis, several key issues need to be addressed to facilitate the design and interpretation of such trials.

1. Cellular heterogeneity: MSC populations vary according to tissue source, donor characteristics, isolation methods, culture conditions, and passage number, which may contribute to variability in therapeutic efficacy.
2. Manufacturing and standardization: Reproducible production, rigorous characterization, potency testing, and quality control remain major challenges for the clinical translation of MSC-based therapies.
3. Delivery and dosing: Optimal routes of administration, cell doses, dosing intervals, tissue targeting, and retention remain incompletely defined and require further investigation.

## 6 Conclusions

IR contributes to atherosclerosis through a complex interplay of endothelial dysfunction, inflammation, dyslipidemia, vascular remodeling, and prothrombotic effects, involving IR-induced trained immunity, tissue selective IR, and mitochondrial dysfunction. Despite substantial evidence implicating these mechanisms, a key question remains unresolved: given the tissue- and pathway-specific nature of insulin signaling, can selective targeting of insulin-signaling pathways attenuate the proatherogenic effects of IR while preserving the beneficial metabolic actions of insulin? Improved understanding of this question may facilitate the identification of novel therapeutic targets. Although stem cells and stem cell derived exosomes appear promising in improving IR in preclinical studies, evidence from clinical trials is limited due to small sample sizes and limited reports on IR improvement. Moreover, a substantial translational gap remains, including the lack of standardized manufacturing protocols and uncertainty regarding optimal routes of administration and dosing. Further studies are warranted to identify tissue- and pathway-specific therapeutic targets and establish the translational feasibility of stem cell- and exosome-based therapies for IR-associated atherosclerotic disease.
